# Modern Endodontics

**DOI:** 10.3390/dj11010011

**Published:** 2022-12-30

**Authors:** Alfredo Iandolo

**Affiliations:** Department of Medicine and Surgery, University of Salerno, 84081 Salerno, Italy; aiandolo@unisa.it; Tel.: +39-3287028233

The goal of modern endodontics is the complete removal of damaged tissue and bacteria from the endodontic space [1,2]. To begin with, this goal can be achieved by performing an accurate diagnosis and using three-dimensional radiographic examinations such as CBCT [3]; a good treatment plan can be organized more precisely.

Currently, with the aid of the use of powerful magnification systems such as an operating microscope and small ultrasonic tips, it is possible to perform access cavities conservatively by retaining more enamel and dentin [4,5]. Afterwards, the mechanical shaping phase of the root canals takes place. The latest generation of rotary files is developed using martensitic alloys, which brings higher flexibility; therefore, the treatment of root canals with accentuated curvatures will be safer and more reproducible [6,7,8].

The endodontic space is a complex area composed of different lateral anatomies such as lateral canals, loops, isthmuses, ramifications, deltas, and dentinal tubules. The shaping phase alone is unable to reach these spaces, which is why active cleansing and 3D cleaning are essential. Once the shaping phase is over, it is important to continue the elimination of bacteria and damaged tissue using irrigants. Indeed, NaOCl and EDTA and their activation protocols will allow the irrigants to work in spaces which files cannot reach. Furthermore, there are several techniques to activate irrigants: subsonic activation, sonic activation, laser, ultrasonic activation, and heat. A highly effective technique that does not require expensive technology is the ultrasonic activation of heated NaOCl [9,10,11].

Finally, the obturation phase comes after completing the chemo-mechanical cleansing of the complex endodontic space. In this phase, gutta-percha cones and sealers are used. In recent years, biosealers have been developed—new-generation endodontic sealers with superior characteristics to traditional sealers [10]. In the event of a failure of an orthograde endodontic treatment, it is possible to save the tooth using surgical endodontics. In addition, new technologies in this field have helped to achieve greater success.

This Special Issue aims to focus on the most modern technologies and protocols, starting from CBCT, access cavities, shaping, active cleansing, and obturation with the latest generation of sealers and surgical endodontics.
